# On the replicability of physiological responses

**DOI:** 10.1242/jeb.250363

**Published:** 2026-03-12

**Authors:** Lesley A. Alton, Candice L. Bywater, Elia Pirtle, Michael R. Kearney, Craig R. White

**Affiliations:** ^1^ School of The Environment, The University of Queensland, St Lucia, QLD 4072, Australia; ^2^ School of Biological Sciences, Monash University, Clayton, VIC 3800, Australia; ^3^ School of BioSciences, The University of Melbourne, Parkville, VIC 3010, Australia

**Keywords:** Metabolic rate, Metabolic depression, Starvation, Food restriction, Plasticity

## Abstract

Science is often claimed to be amid a reproducibility crisis, as evidenced by low replicability of many classic findings across multiple fields. Yet it is not clear how widespread this purported problem is. Physiological responses have the potential for replicability issues because of laboratory-specific biases in animal maintenance as well as technically complex methodologies that are often undertaken using bespoke combinations of equipment. Here, we took advantage of a cross-laboratory manipulative study on metabolic rate to assess the replicability of food restriction effects on metabolic scaling and level. Across seven skink species from the *Egernia* species complex and two universities, we found these responses to be extremely replicable. The slope of the interspecific metabolic scaling relationship was near one and animals reduced their mass-independent rates of energy use by an average of 32% in response to food restriction. This response was consistent across universities. Our study highlights that well designed and replicated studies with a large effect size can indeed be replicable and showcases the value of designing studies that allow tests of replicability to be incorporated explicitly. Such studies will be particularly valuable for treatment effects that generate a small effect size.

## Teaser

**Summary:** The reduction in metabolic rate that accompanies food restriction is replicable across two institutions; suggestions for improving the replicability of physiological research are provided.

## INTRODUCTION

There is lasting concern that science is amid a reproducibility crisis (e.g. Ioannidis, 2005; Open Science Collaboration, 2015; Baker, 2016). Retraction rates of published studies continue to rise (Brembs et al., 2013; Van Noorden, 2023), and the testing of scientific reproducibility is an area of ongoing investigation (e.g. Open Science Collaboration, 2015; Camerer et al., 2016; Camerer et al., 2018; Klein et al., 2018; Clark et al., 2020). Although scientific misconduct is unfortunately a common reason for retraction (Gaudino et al., 2021), and many retractions are attributable to human error (Fang et al., 2012), issues with data and results are also common (Koo and Lin, 2024). Failures of reproducibility might also arise as a consequence of laboratory-specific biases, especially for technically complex measures on laboratory-maintained populations. Indeed, around 25% of reproducibility failures arise as a result of issues with study design (Freedman et al., 2015). A common problem is the failure to replicate at the appropriate scale, such that treatment effects are applied in a way that prevents the effect of treatment from being separated from design elements such as common garden tanks, temperature cabinets or other logistical constraints (for a detailed discussion regarding the principles of experimental design, see Marshall, 2024).

In the present study, we report the results of an experiment designed to test metabolic theory by examining the effect of food restriction on the scaling of metabolic rate (see table 1, test 10 of Kearney and White, 2012; see also White et al., 2022; White and Marshall, 2023). The measurement of metabolic rate is highly technical and there is growing awareness and concern about its replicability (Corrigan et al., 2020; Killen et al., 2021; Wu et al., 2024; Banks et al., 2025). Here, we consider the findings of our study purely in the context of replicability; the theoretical implications of our findings will be discussed elsewhere. Undertaking such an experiment required us to consider how best to deploy resources across multiple institutions, to ensure that we applied our treatment (food restriction) at the appropriate scale (Marshall, 2024). We collected data for seven species of skink from the *Egernia* species complex (While et al., 2015); for those species for which we had a sufficiently large number of individuals (five species), we housed and measured them at both The University of Queensland and The University of Melbourne. We took this approach to avoid repeating common replication problems by ensuring that we did not confound treatment with housing location. This approach presented us with the opportunity to determine whether the strength and magnitude of responses to food restriction were consistent across institutions despite unavoidable differences in food source, housing and measurement systems at each location. Following the Claerbout/Donoho/Peng terminology summarised by Barba (2018 preprint), we refer to this as a replication study (collection of new data by separate teams, possibly with different methods) even though the data were analysed together (this approach is distinct from the related concept of reproducible research, where authors provide all the necessary data and the computer codes to run the analysis again, re-creating the results).

## MATERIALS AND METHODS

Some elements of the methods for collection, housing and measurement have been presented elsewhere (Wu et al., 2015; White et al., 2019). We repeat them here in full, so that differences between locations are explicit.

### Animal collection

Skinks were obtained from a range of sources between October 2011 and May 2012. They were either collected from the wild at various locations across Australia or purchased from recreational breeders located in south-eastern Queensland. Skinks purchased from recreational breeders were predominantly juveniles that were less than 6 months old, whereas wild-caught individuals were predominantly adults given their size. Skinks collected from the wild were originally maintained at The University of Melbourne (UM) while skinks purchased from breeders were originally maintained at The University of Queensland (UQ). In August 2012, individuals of five species [desert skink, *Liopholis inornata*; (Rosén 1905); night skink, *Liopholis striata* (Sternfeld 1919); tree-crevice skink, *Egernia striolata* (Peters 1870); Cunningham's skink, *Egernia cunninghami* (Gray 1832); blue-tongued skink, *Tiliqua scincoides* (White 1790)] were randomly divided between UM and UQ such that the two universities had approximately equal numbers of all species. In March 2014, three major skinks, *Bellatorias frerei* (Günther 1897), and four pink-tongued skinks, *Cyclodomorphus gerrardii* (Gray 1845), that were originally obtained from breeders as juveniles were returned to UQ from UM for food restriction and metabolic rate measurements. Skinks were collected and housed under appropriate permits (Government of Western Australia Department of Environment and Conservation Licence SF008358; Victoria Department of Sustainability and Environment Permit 10005993; Queensland Department of Environment and Resource Management Scientific Purposes Permit WISP10698712), and handled and measured in accordance with animal ethics approvals from The University of Queensland (Certificate SBS/288/11/ARC) and The University of Melbourne (Ethics ID 1112194).

### Animal maintenance

Skinks were housed individually in white plastic containers (600 mm×400 mm×260 mm, length×width×depth) in temperature-controlled rooms that were maintained at 20±5°C. Each container was positioned under two linear fluorescent light bulbs that provided a 12 h:12 h light:dark photoperiod. One light bulb emitted visible, ultraviolet-A and ultraviolet-B radiation (Repti Glo 10.0, Exo Terra, Montreal, QC, Canada), and was replaced annually, while the other emitted only visible radiation (Crompton Lighting, Padstow, NSW, Australia). To allow skinks to thermoregulate behaviourally, they were provided with a black plastic refuge that was positioned at one end of the container directly under a 50 W halogen lamp that heated the top of the refuge to 35±2°C, the inside of the refuge to 30±2°C and the opposite end of the container to 25±2°C for 8 h in the middle of the 12 h light cycle. Black sandpaper was also affixed to the top of the refuge to help with general animal health, particularly skin shedding and nail length. The mean relative humidity in the containers was maintained at approximately 60% by keeping a sponge saturated with water inside each of the containers. All species were maintained on a substrate of paper, except *L. inornata* and *L. striata*, which were provided with a sand substrate that was 30 mm deep. Containers with a paper substrate were cleaned with soapy water, and the paper replaced, twice a week. Containers with a sand substrate had faeces removed twice a week and the sand replaced every 6 months.

Skinks were given access to water at all times and were fed twice a week on a diet of finely processed raw food that consisted of turkey mince and/or beef mince, butternut pumpkin, green beans, strawberries and rocket. At UM, the mass ratio of ingredients was 2:2:0.5:0.25:0.1, respectively, with a 1.5:0.5 mass ratio of turkey to beef mince. At UQ, the mass ratio of ingredients was 2:1.75:0.75:0.5:0.1, respectively, and beef mince was used only when turkey mince was unavailable. This diet was supplemented with a reptile-specific multivitamin (Herptivite™, Rep-Cal, Los Gatos, CA, USA) and calcium powder (phosphorus-free calcium with vitamin D3 Ultrafine, Rep-Cal) at the recommended dose of 15 ml of each supplement per 1 kg of food.

### Food restriction

The objective of the food restriction protocol was to generate a plane of nutrition that resulted in a 15% reduction in body mass. Animals assigned to the control group continued to have food provided twice a week, while a smaller volume of food was given to food-restricted animals once a week at UM and twice or once a week at UQ. At UM, animals were maintained on these feeding regimes until food-restricted animals had lost 15% of their body mass relative to their starting mass prior to food restriction, and this was also the case for two food-restricted blued-tongued skinks at UQ that showed consistent mass loss. However, for other food-restricted animals at UQ, the extent of their mass loss was made relative to a non-food-restricted animal of the same structural size (estimated as the first principal component, PC1, of a principal component analysis that included snout–vent length, tail length and head length). To estimate the mean mass loss of food-restricted animals for each species at UQ, a linear model was fitted to body mass data with PC1 (i.e. structural size) as a continuous co-variate and feeding treatment (control or food restricted) as a fixed factor. The parameter estimate for treatment was used to calculate the percentage change in the intercept of the relationship between body mass and structural size for control and food-restricted animals, and once this was equal to −15% the metabolic rates of the animals was measured.

### Respirometry: UQ

Following food restriction, the metabolic rate of each skink was estimated by measuring their rate of O_2_ consumption or CO_2_ production using positive-pressure open-flow respirometry. Prior to measurements, skinks were fasted for between 4 and 11 days to ensure that they were postabsorptive. All measurements were made at 25±1°C in a temperature-controlled room. Atmospheric air was drawn from outside using a pump (TR-SS3, Sable Systems International, North Las Vegas, NV, USA) and scrubbed of CO_2_ using soda lime and water vapour using Drierite before passing through a mass flow controller (GFC17, Aalborg, Orangeburg, NY, USA) that maintained flow rates at levels appropriate for the species of different size. Mass flow controllers were calibrated using a NIST-traceable bubble flow meter (1–10–500 ml; Bubble-O-Meter, Dublin, OH, USA). Air then passed through an appropriately sized custom built respirometry chamber containing the animal. The outflow of air from the respirometry chamber was scrubbed of water vapour using Drierite before passing through a CO_2_ analyser (LI-7000, LI-COR, Lincoln, NV, USA) and an O_2_ analyser (Oxzilla II, Sable Systems International). The CO_2_ and O_2_ analysers were interfaced with a PowerLab 8/30 A/D convertor (ADInstruments, Bella Vista, NSW, Australia), which recorded fractional concentrations of CO_2_ and O_2_ in the excurrent air at a frequency of 10 Hz. The CO_2_ analyser was calibrated with dry CO_2_-free air and a certified gas mixture (0.386±0.008% CO_2_ in N_2_; BOC Gases, Wetherill Park, NSW, Australia), and the O_2_ analyser was calibrated using dry CO_2_-free air. Rates of O_2_ uptake and CO_2_ production were calculated using standard equations (Lighton, 2008) for the lowest 3 h interval during the measurement period of 12–18 h.

### Respirometry: UM

Flow-through respirometry was used to measure the rate of evaporative water loss, as well as the rate of CO_2_ production and O_2_ consumption, which were used as proxies for metabolic rate. Air was drawn from a compressed air source via an intermediate 15 l reservoir using a Sub-Sampler Pump (SS-4, Sable Systems International) and scrubbed of CO_2_ and water vapour via sequential columns of soda lime (Chem Supply granular 4–10 mesh, UM Chemstore) and Drierite (2 mm granule, 8 mesh, Labchem Inc., Thermo Fisher Scientific Australia Pty Ltd, Scoresby, VIC, Australia), respectively. The airflow was then split and a Mass Flow Control Unit (MFC2, Sable Systems International) was used to control the flow rate through two mass flow control valves (Sierra Instruments, flow capacity of 200 ml min^−1^ and 1000 ml min^–1^, 0°C, 1 atm, calibrated for air/N_2_) allowing for separate, concurrent, control and animal measurements in custom­-built species-specific respirometry chambers. Excurrent air passed through a water vapour analyser (RH-300, Sable Systems International), and was then scrubbed of water vapour using a Drierite column, before passing through a CO_2_ analyser (CA-10A, Sable Systems International), then scrubbed of remaining CO_2_ and water vapour using a column of soda lime and Drierite, respectively, before passing through a differential O_2_ analyser (Oxilla II, Sable Systems International). Flow controllers and gas analysers were interfaced with an A-D converter (Universal Interface UI-2 Eight channel, 16-bit Data Acquisition Interface, Sable Systems International) and data were recorded once every second to a computer running LabHelper X (Warthog Systems, Mark A. Chappell, University of California, Riverside, USA). Flow rate through the chamber was recorded concurrently and varied depending on the species being measured and ranged between 100 and 200 ml min^−1^. Flow meters were calibrated using a NIST-traceable bubble flow meter (1–10–500 ml; Bubble-O-Meter). Each individual was measured overnight for a minimum of 18.5 h which allowed for 3×3.5 h animal measurements and 4×2 h baseline measurements. Control and animal chambers, both water vapour units and the multiplexer were housed within a temperature controlled cabinet (Euotherm 3500 Series) at 25±1°C. All remaining analysers were housed outside the cabinet at 24±2°C. Food was withheld from animals for a minimum of 48 h prior to being measured to ensure a post-absorptive state and to minimise the risk of faeces and urine in the chamber. Respirometry traces were analysed using LabAnalyst X (Warthog Systems, Mark A. Chappell, University of California, Riverside, USA). Prior to analysis, raw data were baseline and lag corrected. Rates of O_2_ uptake and CO_2_ production were calculated using standard equations (Lighton, 2008) for the lowest (O_2_ consumption) 2.5 h interval during the measurement period.

### Statistical analysis

Data for standard metabolic rate (SMR) were first analysed using a Bayesian phylogenetic linear mixed model (PMM) implemented in the *brms* package (Bürkner, 2017, 2018, 2021) of R v4.4.1 (http://www.R-project.org/). Phylogenetic relatedness was estimated using a dated phylogeny for social skinks (Scincidae: Egerniinae) based on an analysis of combined continuous morphological, discrete morphological and molecular characters (Thorn et al., 2019). We first log_10_-transformed and centred both body mass (*M*, g) and metabolic rate (MR, ml h^–1^), and tested the effect of food restriction across all species using a model that included main effects for log_10_*M*, treatment (control or food restricted) and institution (UM or UQ), as well as their two- and three-way interactions; categorical fixed effects were coded using sum-to-zero contrasts. The model also included random intercepts for phylogeny and species identity, and random slopes for the effect of treatment, university and log_10_*M* as a function of species identity. The model was run with default priors, five chains, 40,000 iterations, a warm-up of 20,000 samples and a thinning interval of 100 samples. This yielded R-hat (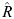) values less than 1.01, effective samples sizes greater than 880, and a posterior distribution with 1000 samples. To understand the importance of the random effects, we calculated the proportion of variance, conditioned on the fixed effects, associated with each of the random intercept and slope terms. We then used the parameter estimate for the effect of log_10_*M* from this model to adjust metabolic rate for each species to a common body mass equal to the mean body mass of all individuals of that species.

We next used a multilevel meta-analysis to determine whether the treatment effect of food restriction varied among universities, using Hedges' *g* as a measure of standardised mean difference. This analysis was undertaken using the *metacont* function of the R package *meta* (Balduzzi et al., 2019). We interpret effect size as being small (*g*=0.2), medium (*g*=0.5) or large (*g*≥0.8) (Cohen, 1988).

Finally, we pooled data from both institutions and used Bayesian PMMs implemented using Integrated Nested Laplace approximation in the R package INLA v24.12.11 (Rue et al., 2009, 2017; Martins et al., 2013) to estimate the scaling exponent of SMR for control and food-restricted animals. We first fitted a model that included centred log_10_MR as a response and centred log_10_*M* and treatment as predictors, plus their interaction; this model included random intercept terms for phylogeny and species identify independent of phylogeny. We then estimated the scaling relationship between (non-centred) log_10_MR and (non-centred) log_10_*M* for control and food-restricted animals separately, in a model that included a random intercept term for phylogeny; the parameter estimates for these last two models were used for plotting.

## RESULTS

**Fig. 1.**
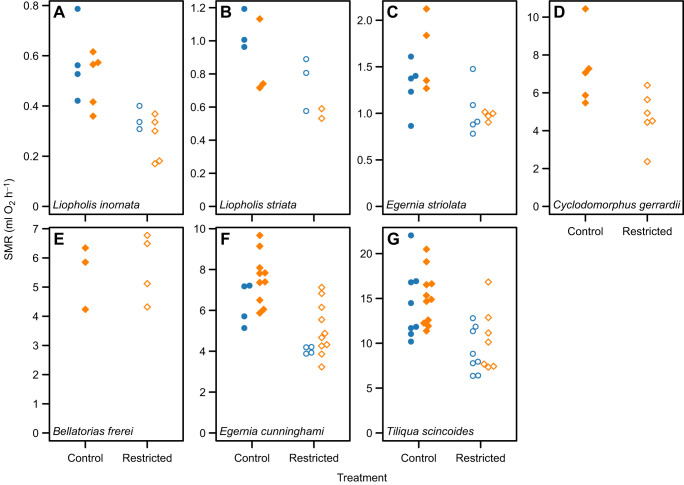
**The effect of food restriction on the standard metabolic rate (SMR) of skinks from the *Egernia* species complex.** (A) *Liopholis inornata*, (B) *Liopholis striata*, (C) *Egernia striolata*, (D) *Cyclodomorphus gerrardii*, (E) *Bellatorias frerei*, (F) *Egernia cunninghami* and (G) *Tiliqua scincoides*. Data collected at The University of Melbourne (UM) are shown as blue circles and data collected at The University of Queensland (UQ) are shown as orange diamonds. Filled symbols denote control animals and open symbols denote food-restricted animals. Data for SMR were adjusted for variation in body mass as described in Materials and Methods.

**Fig. 2.**
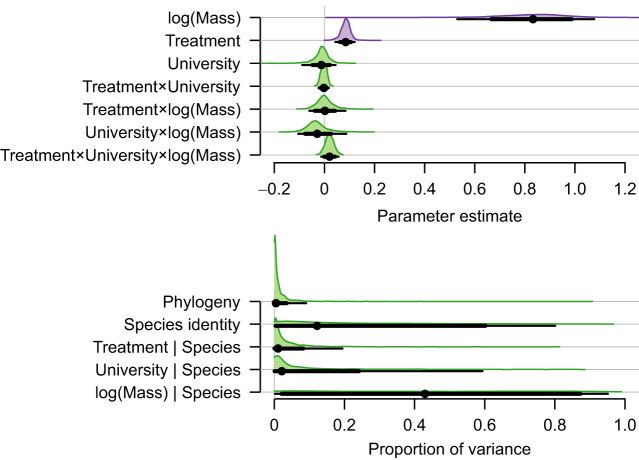
**The effect of food restriction, body size and university across all species.** Posterior distributions, 80% credible interval (CI; thick black line), 95% CI (thin black line) and mean (filled points) for the fixed and random effect for a model that described SMR as a function of the fixed effects of body mass, university (UM or UQ) and treatment (control or food restricted), and a full factorial combination of their interactions. The model included a random intercept for phylogeny, and random slopes for university, mass and treatment for the effects of species identity independent of phylogeny. SMR and mass were log_10_-transformed and centred. The fixed effects of mass and treatment were significant and are shown in purple; all other fixed effects and interactions were not significant and are shown in green.

Data for body mass and SMR for each of the species and institutions are summarised in Table 1 and data for individuals of each species are plotted in Fig. 1. When data for all species were considered together, no interactions were significant (Fig. 2). The parameter estimate (±s.e.) for the effect of log_10_*M* (the mean within-species scaling exponent) was 0.88±0.06 and the parameter estimate for the effect of treatment (food restriction) was 0.085±0.011. The SMR of food-restricted animals was therefore equal to 0.68 of the control animals (95% highest posterior density interval: 0.61, 0.75).

**Table 1.** Summary statistics for body mass and standard metabolic rate (SMR) for animals housed and measured at The University of Melbourne (UM) and The University of Queensland (UQ) under control (C) and food-restricted (F) treatment conditions

Uni.	Species	Treatment	Body mass (g)			SMR (ml h^−1^)			*n*
			Mean	s.d.	s.e.m.	Mean	s.d.	s.e.m.	
UM	*Egernia cunninghami*	C	236	76	38	7.11	1.77	0.88	4
UM	*Egernia cunninghami*	F	237	76	38	4.65	1.05	0.53	4
UM	*Egernia striolata*	C	37.7	2.1	0.9	1.36	0.30	0.13	5
UM	*Egernia striolata*	F	30.7	2.7	1.2	0.91	0.31	0.14	5
UM	*Liopholis inornata*	C	13.7	1.0	0.5	0.62	0.16	0.08	4
UM	*Liopholis inornata*	F	12.1	2.8	1.6	0.35	0.11	0.07	3
UM	*Liopholis striata*	C	30.9	2.5	1.5	1.18	0.17	0.10	3
UM	*Liopholis striata*	F	23.6	3.9	2.2	0.671	0.139	0.081	3
UM	*Tiliqua scincoides*	C	521	74	26	17.2	5.9	2.1	8
UM	*Tiliqua scincoides*	F	379	37	13	8.25	2.01	0.71	8
UQ	*Bellatorias frerei*	C	142	19	11	5.92	0.73	0.42	3
UQ	*Bellatorias frerei*	F	119	22	11	5.35	1.24	0.62	4
UQ	*Cyclodomorphus gerrardii*	C	127	48	21	8.07	3.86	1.72	5
UQ	*Cyclodomorphus gerrardii*	F	114	48	20	4.85	2.66	1.08	6
UQ	*Egernia cunninghami*	C	216	54	17	8.13	2.26	0.72	10
UQ	*Egernia cunninghami*	F	175	56	18	4.45	1.24	0.39	10
UQ	*Egernia striolata*	C	39.3	5.8	2.9	1.79	0.59	0.29	4
UQ	*Egernia striolata*	F	37.8	6.2	3.1	1.01	0.10	0.05	4
UQ	*Liopholis inornata*	C	13.1	1.2	0.6	0.527	0.110	0.049	5
UQ	*Liopholis inornata*	F	11.2	1.6	0.7	0.249	0.088	0.039	5
UQ	*Liopholis striata*	C	28.3	4.3	2.5	0.879	0.121	0.070	3
UQ	*Liopholis striata*	F	25.8	1.9	1.4	0.542	0.073	0.052	2
UQ	*Tiliqua scincoides*	C	451	125	38	15.7	4.8	1.5	11
UQ	*Tiliqua scincoides*	F	383	81	31	9.33	2.41	0.91	7

**Fig. 3.**
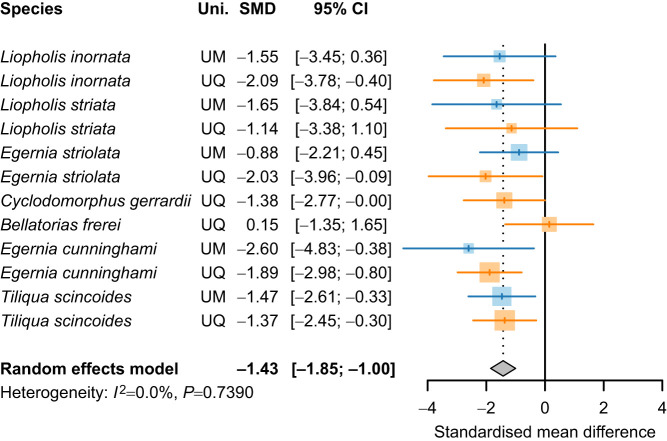
**Estimates of the effect size of feeding treatment for each of the species-by-university combinations, as well as the meta-analysis result.** Standardised mean difference (SMD) of the effect size of the feeding treatment is shown (Hedges *g*, shown ±95% CI). Grey diamond indicates the meta-analysis result, where the treatment estimate is represented by the centre of the diamond and the width of the diamond represents the 95% CI. For each species-by-university combination, the coloured vertical line represents the effect size and the size of the coloured square represents the precision of individual treatment estimates based on the random effects meta-analysis (i.e. larger squares represent more precise estimates of the effect of treatment). Data collected at UM are shown in blue; data collected at UQ are shown in orange.

The multilevel meta-analysis revealed that the magnitude of the effect of food restriction was large (*g*=−1.42, *P*<0.001; Fig. 3). Heterogeneity was negligible both within universities (τ^2^<0.001, 95% CI: 0, 0.62) and between universities (τ^2^<0.001; 95% CI: 0, 4.6). The proportion of variance due to heterogeneity was not significantly different from zero (*I*^2^=0.0%, *Q*_11_=7.68, *P*=0.74).

**Fig. 4.**
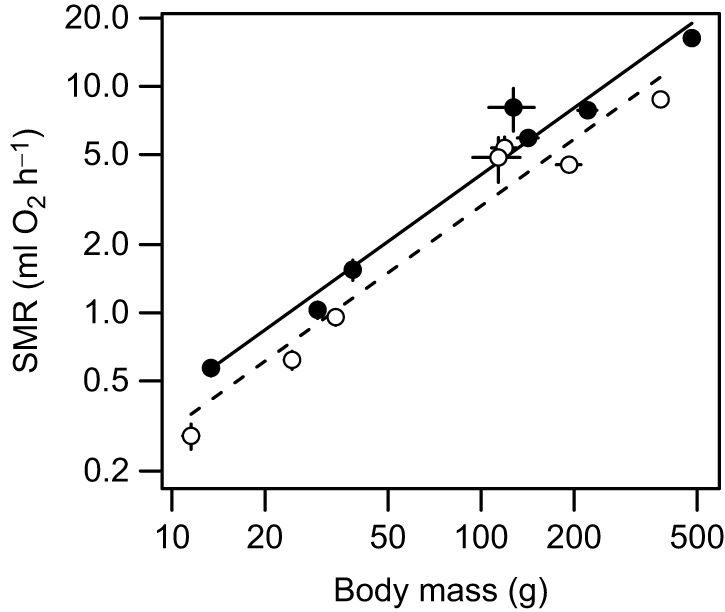
**The among-species scaling of SMR with body mass for seven skink species of the *Egernia* species complex.** Data were collected at UM and UQ and combined, and are shown as means±s.e.m. for control animals (solid line and filled circles) and food-restricted animals (dashed line and open circles). Data for SMR and body mass are each plotted on a log scale.

When the data collected at the two institutions were pooled, there was no significant interaction between scaled log_10_*M* and treatment (parameter estimate: –0.22±0.06; 95% CI: −0.142, 0.098) and a significant effect of treatment (parameter estimate: 0.073±0.03; 95% CI: 0.012, 0.133). The among-species scaling exponent of metabolic rate was 0.98±0.07 for control animals and 0.98±0.11 for food-restricted animals (Fig. 4).

## DISCUSSION

In contrast to revelations that a large proportion of findings cannot be replicated (e.g. Freedman et al., 2015; Open Science Collaboration, 2015; Baker, 2016; Camerer et al., 2016, 2018; Klein et al., 2018; Clark et al., 2020), we found consistent physiological responses to food restriction across two universities and multiple species. This was despite known inconsistencies in methodological details across laboratory settings (i.e. the source of food, the specifics of the food restriction end point, and the respirometry system used to measure metabolic rate).

Previous work on the replicability of metabolic rate measurements indicates that it is repeatable for individual animals but that this repeatability declines through time (Nespolo and Franco, 2007; White et al., 2013), and that it is heritable (Pettersen et al., 2018). However, we are unaware of assessments of replicability of metabolic phenomena across laboratories. The main objective of our study design was to ensure that we did not confound food restriction or species identity with institutional setting. Our finding that physiological responses can be replicable is reassuring, and perhaps surprising, given the technical complexity of the measurements and the large potential for effects due to unavoidable differences in animal husbandry at the different institutions. The approach we adopted is similar to within-study replication (where the same research team repeatedly initiates an experiment in multiple years and/or locations) and distributed network replication (where experiments with standardised protocols are run concurrently by multiple research groups in multiple locations). Both of these approaches have been proposed to improve the confidence of studies in field ecology (Fraser et al., 2013; Filazzola and Cahill, 2021), and we show here that such approaches can also improve confidence in studies of animal physiology.

We conclude by offering suggestions for the factors that should be considered to achieve increased replicability: experimental design, effect size and sample size. To maximise replicability, it is essential to design experiments where replication occurs at the appropriate biological scale and that factors of interest are not confounded (Marshall, 2024). Animals were housed individually in the present study, and the treatment could therefore be applied at the level of the individual. But it is not always practical to apply treatments at the level of the individual. Treatments are therefore often applied at a higher scale, such as at the scale of tanks or cabinets that each contain multiple individuals. When the treatment is applied at the scale of the tank or cabinet, individual animals are no longer replicates. Such designs are common in animal physiology, and our own work provides numerous examples of this practice (e.g. Schimpf et al., 2009; Vaca and White, 2010; Crispin and White, 2013; Bartrim et al., 2014; Winwood-Smith et al., 2015; Turschwell and White, 2016; Callaghan et al., 2021; Tan et al., 2023; Alton et al., 2024; Orford et al., 2024; Schofield and White, 2025). If experiments are not replicated at the scale at which the treatment is applied (i.e. if each treatment level does not include replicate tanks or cabinets), then tank or cabinet will add (unknown and unquantifiable) noise and thereby result in the misestimation of effect sizes.

Larger effect sizes are more replicable (Boyce et al., 2023; Li et al., 2024). The replicable effect observed in the present study, for example, is large: food restriction reduced SMR by an average of 32% with a large overall effect (Hedges' *g*=−1.43). Our sample size was also quite large; 126 measurements across all species (Table 1). Sample size is a significant predictor of replicability, for a given effect size (Li et al., 2024). Appropriate consideration should therefore also be given to sample size when designing experiments (e.g. McNab, 2003; Duffy et al., 2021), especially when the anticipated effect size is small. Sample sizes per group required to achieve a statistical power of 0.8 for effect sizes of 0.2, 0.5 and 0.8 in a two-sample *t*-test are 394, 64 and 26, respectively (calculated using the *pwr.t.test* function of the *pwr* package of R: https://CRAN.R-project.org/package=pwr). Such effect sizes would correspond with reductions in metabolic rate of around 5%, 12.5% and 20% in the present study, based on the median observed coefficient of variation (CV=s.d./mean) (25%: Table 1). Designing experiments that have sufficient power to detect effect sizes of interest for relevant traits will help to ensure replicability. As one example, it is necessary to measure thousands of individuals to ensure the replicability of brain–behavioural phenotype associations established using magnetic resonance imaging (Marek et al., 2022). Large sample sizes will also serve to reduce the risk of Type II errors (failure to reject the null hypothesis when it is false) (Jennions and Møller, 2003) and sign errors (Parker et al., 2016), and reduce the risk of succumbing to the ‘winner's curse’, where studies finding a significant effect with a small sample size are likely to overestimate the effect size (Lemoine et al., 2016).
